# Genetics of migraine in the age of genome-wide association studies

**DOI:** 10.1007/s10194-011-0399-0

**Published:** 2011-11-11

**Authors:** Markus Schürks

**Affiliations:** Department of Neurology, University Hospital Essen, Hufelandstrasse 55, 45122 Essen, Germany

**Keywords:** Migraine, Migraine with aura, Genetics, Genome-wide association studies, Glutamate, *TRPM8*, *PRDM16*, *LRP1*

## Abstract

Genetic factors importantly contribute to migraine. However, unlike for rare monogenic forms of migraine, approaches to identify genes for common forms of migraine have been of limited success. Candidate gene association studies were often negative and positive results were often not replicated or replication failed. Further, the significance of positive results from linkage studies remains unclear owing to the inability to pinpoint the genes under the peaks that may be involved in migraine. Problems hampering these studies include limited sample sizes, methods of migraine ascertainment, and the heterogeneous clinical phenotype. Three genome-wide association studies are available now and have successfully identified four new genetic variants associated with migraine. One new variant (rs1835740) modulates glutamate homeostasis, thus integrates well with current concepts of neurotransmitter disturbances. This variant may be more specific for severe forms of migraine such as migraine with aura than migraine without aura. Another variant (rs11172113) implicates the lipoprotein receptor LRP1, which may interact with neuronal glutamate receptors, thus also providing a link to the glutamate pathway. In contrast, rs10166942 is in close proximity to *TRPM8*, which codes for a cold and pain sensor. For the first time this links a gene explicitly implicated in pain related pathways to migraine. The potential function of the fourth variant rs2651899 (*PRDM16*) in migraine is unclear. All these variants only confer a small to moderate change in risk for migraine, which concurs with migraine being a heterogeneous disorder. Ongoing large international collaborations will likely identify additional gene variants for migraine.

## Introduction

Migraine is a common neurological disorder affecting between 10 and 20% of the population [1]. The clinical presentation is heterogeneous and includes recurrent headache attacks, associated symptoms of vegetative disturbance, and hypersensitivity of various functional systems of the nervous system [1, 2]. About one-third of migraineurs further experience transient neurological symptoms mostly involving the visual system prior to or during a migraine attack, which are known as migraine aura [2]. The International Headache Society (IHS) has established gold-standard criteria for the diagnosis of migraine [3]. While migraines with aura (MA) and without aura (MO) are the most common forms, the IHS also acknowledges, for example, certain forms lacking one diagnostic criterion as probable migraine as well as rare familial monogenetic forms.

Heredity has been shown to play an important role in migraine pathogenesis. About 50% of affected individuals have a first-degree relative also suffering from migraine [4–6]. In addition, family and twin studies support the idea of MO and MA being different phenotypes of the same entity, with a heritability ranging from 33 to 57% [5, 7, 8].

The broad clinical spectrum of migraine and the results from heritability studies have posted a challenge to the understanding of the underlying pathophysiology and molecular genetics. For example, it is difficult to find a pathophysiological link between a headache, which is not symptomatic, and symptoms like nausea, photophobia, or hypotension. In addition, it is unclear if some of the clinical symptoms are more genetically determined and some are more environmentally determined. On the contrary, dissecting the migraine phenotype into endophenotypes has offered opportunities to better understand the pathophysiological processes involved, facilitating the formulation of hypotheses for genetic studies. For example, symptoms like yawning (premonitory phase) and nausea (premonitory and headache phase) point toward an involvement of dopamine and have entailed genetic association studies of variants in genes involved in dopamine metabolism [9]. Another approach was to utilize epidemiological data for hypothesis generation. The three- to fourfold higher prevalence of migraine among women than men [10] as well as attacks following a menstrual cycle pattern [11] suggested an involvement of sex hormones and led to the investigation of genetic variants in sex hormone receptor genes [12]. Aware of the limitations of these approaches, methods free of assumptions have been employed mining the genome for associations with the migraine phenotype or certain migraine symptoms. These include linkage studies and genome-wide association studies (GWAS). In contrast to all approaches focusing on common forms of migraine, investigations in rare familial forms of migraine, presenting with a well circumscribed aura in the form of a hemiparesis/hemiplegia (familial hemiplegic migraine, FHM), have been more successful in identifying underlying genetic causes [13–15].

This manuscript does not aim at providing a complete overview of genetic studies in migraine; this has comprehensively been done recently [16, 17]. This manuscript will provide examples for different approaches used in migraine genetics and then focus on results from the recently published GWASs.

## Familial hemiplegic migraine (FHM)

FHM is a rare, but severe form of migraine displaying an autosomal dominant mode of transmission. The main characteristic of FHM is a prolonged aura in the form of a hemiparesis/hemiplegia presenting with the migraine headache. FHM belongs to the group of channelopathies and multiple genetic variants in three different genes coding for neuronal ion channels have been identified as causative. These genetic variants perturb the activity of the channels, thus increasing neuronal excitability. In FHM type 1 the affected gene is *CACNA1A* (chromosome 19p13), coding for a P/Q calcium channel [15], in FHM type 2 the *ATP1A2* gene (chromosome 1q23) is affected, encoding a subunit of the Na^+^/K^+^-ATPase [13], and FHM type 3 is caused by variants in the *SCN1A* gene (chromosome 2q24), coding for a sodium channel [14]. However, patients with FHM only constitute a very small proportion of all migraineurs and there is little evidence, that the identified genes play a major role in common forms of migraine [18]. This may also suggest that the ion channel genes are only important for the aura and not the migraine per se, since, in particular, the *CACNA1A* gene is also implicated in other neurological disorders not associated with migraine including episodic ataxia type 2 and spinocerebellar ataxia type 6.

## Candidate gene approaches

Candidate gene approaches have been a popular tool in studying migraine genetics. Genes investigated are, for example, implicated in serotonin and dopamine metabolism, neurogenic inflammation, vascular function, and hormone regulation.

Migraine is viewed as an inherited brain disorder, characterized by neurotransmitter imbalances that lead to neuronal dysfunctions [19]. The serotonergic system plays an important role and alterations in serotonin metabolism and in the processing of central serotonin-mediated responses are typical features among migraineurs [20]. Following axonal release serotonin (5-HT) is rapidly taken up again by the presynaptic serotonin transporter SLC6A4 (5-HTT) [21]. An insertion/deletion polymorphism in the promoter region of the SLC6A4 gene, termed 5-HTTLPR [22, 23] has functional impact on the synaptic clearing of serotonin [24]. Ten studies in different populations have investigated the association between the *5*-*HTTLPR* gene and migraine among Europeans and Asians [25]. Pooled analyses did not find an overall association; however, there was some suggestion that gender and migraine aura status may have modifying roles among Europeans.

Another neurotransmitter implicated in migraine pathogenesis is dopamine. Symptoms like yawning in the premonitory phase and nausea in both the premonitory and headache phase are ascribed to dopamine [26]. There is also evidence from other pain disorders that dopamine plays a role in central pain [27]. Further support for a role of dopamine in migraine comes from studies showing that anti-dopaminergic drugs exert beneficial effects, when administered during migraine attacks and from experimental work showing that dopamine modulates the neuronal firing rate in the trigeminocervical complex [9]. Genetic association studies have mostly investigated variants in dopamine receptor genes and the dopamine beta-hydroxylase gene; however, study results are conflicting and the significance of these variants in migraine remains unclear.

A “neurogenic inflammation” appears to be a key mechanism in causing the migraine headache by activating the trigeminal system [28]. Tumor necrosis factor-alpha (TNF-alpha) is an important inflammatory cytokine and modulator of immune responses. Its role in migraine has been postulated based on reports of TNF-alpha concentration changes in serum [29–31] and urine [32] among migraineurs, as well as findings that TNF-alpha can stimulate transcription of calcitonin gene-related peptide (CGRP), which is pivotal in migraine pathophysiology [33]. Variants in the genes coding for TNF-alpha and the closely related TNF-beta have been shown to modulate cytokine levels of TNF-alpha and TNF-beta [34, 35] prompting investigations of various polymorphisms in these genes with respect to their role in migraine. Among the ten studies investigating variants in these genes, the best evidence is available for the *TNF*-*alpha* -308G>A and *TNF*-*beta* 252A>G polymorphisms. Pooled analyses did not indicate an overall association with migraine [36]. While subgroup analyses suggested that associations may differ among populations of non-Caucasian origin, these findings only stem from few studies.

Migraine prevalence is three- to fourfold higher in women than men [10]. Further, some women suffer from migraine attacks presenting at specific times during the menstrual cycle or experience changing patterns of attacks during pregnancy or after menopause [11, 37]. Finally, changes in estrogen levels can trigger migraine attacks [11, 37]. These findings have prompted studies investigating variants in genes coding for proteins in sex hormone receptor pathways and metabolism among migraineurs. Pooled analyses of the available studies suggest that the estrogen receptor-1 gene (*ESR*-*1*) 594 G>A and 325 C>G polymorphisms can modify the risk for migraine, a finding that does not differ between MA and MO [12]. In contrast, the *ESR*-*1* Pvu II C>T polymorphism and the progesterone receptor (*PGR*) PROGINS insert polymorphism do not appear to be associated with migraine.

The vasculature appears to play a special role in migraine. For example, migraineurs carry an approximately twofold increased risk for ischemic stroke compared to non-migraineurs [38], some vasculopathies are frequently observed among migraineurs such as livedo reticularis [39] and Sneddon’s syndrome [40], and altered vascular reactivity can be found among young migraineurs even in the absence of other disorders [41]. These findings may be linked to oxidative stress causing endothelial dysfunction [42]. Specifically, certain gene variants in the methylenetetrahydrofolate reductase gene (*MTHFR* 677C>T polymorphism) and angiotensin-converting enzyme gene (*ACE* D/I polymorphism) appear to importantly contribute to the vascular oxidative stress response [42]. Consequently, an association between the *MTHFR* 677C>T and *ACE* D/I polymorphisms with migraine has been suggested [42]. Both are functional variants. The *MTHFR* 677TT genotype impairs enzyme activity and carriers have increased homocysteine levels. Carriers of the *ACE* II genotype have ACE plasma levels half that of DD subjects, with ID subjects having intermediate levels. Association studies investigating the role of these variants in migraine were controversial. Pooled analyses of 13 studies indicated that the *MTHFR* 677TT genotype was associated with a 48% increased risk for MA (pooled odds ratio [OR] = 1.48, 95% confidence interval [CI] 1.02–2.13), but not MO [43]. In contrast, pooled results from nine studies indicated that the *ACE* II genotype was associated with a reduced risk for MA (pooled OR = 0.71, 95% CI 0.55–0.93) and MO (pooled OR = 0.84, 95% CI 0.70–0.99) [43].

## Genome-wide approaches

In contrast to candidate gene association studies, genome-wide approaches have been employed to identify potential new chromosomal regions or genes that may associate with migraine, migraine subtypes, or migraine features. These are basically data mining techniques screening a part or the whole genome. The two main methods employed are linkage analyses that study pedigrees of migraineurs and GWAS that investigate large populations of migraineurs and non-migraineurs.

## Linkage studies

Linkage analyses have identified several genetic loci for overall migraine, MA, and MO. However, the underlying genes from these chromosomal regions that may plausibly alter risk of migraine have not yet been identified. Overall migraine has been linked to chromosomes Xq24-28 [44], 6p12.2-21.1 [45], 19p13 [46], and 1q31 [47]; MA to 4q24 [48], 9q21-q22 [49], 11q24 [50], and 15q11-13 [51]; and MO to 14q21.1-22.3 [52], and 4q21 [53].

## Alternative phenotyping methods

Only two independent linkage studies from Iceland and Finland describe an overlapping gene locus on chromosome 4 [48, 53]. However, the other studies have reported many different loci, which is likely a reflection of the genetic heterogeneity of migraine. The heterogeneous migraine phenotype with a broad spectrum poses a particular challenge to genetic studies, which may in part explain the genetic heterogeneity observed. Hence, more clearly defined individual features of migraine (“endophenotypes”) may help to more consistently identify genetic loci or genes involved in migraine [54]. For example, employing trait component analysis and latent class analysis has successfully linked certain chromosomal regions to pulsating pain, phono-/photophobia, nausea, and age at onset of migraine [44, 55, 56].

## Genome-wide association studies

The first GWAS in migraine was published by the International Headache Genetics Consortium (IHGC) in 2010 [57] (Table 1). This was a two-stage association study involving seven clinic-based case collections. In the discovery stage 2,731 migraineurs all suffering from MA, recruited from clinics in Finland, Germany, and the Netherlands, were compared with 10,747 population-based controls from the same countries. In the replication stage an additional 3,202 cases from clinics in Denmark, the Netherlands, and Germany as well as from the general population in Iceland were available and compared to 40,062 population-based controls. Patients used for replication suffered either from MA or MO. Diagnoses were made by headache experts using a combination of questionnaires and direct interviews based on the 2004 IHS diagnostic criteria [3]. The following diagnostic subgroups were analyzed: overall migraine, MA only, MA and MO, and MO only.

**Table 1:** Overview of published GWAS in migraine

Study	Anttila et al. [57]	Ligthart et al. [61]	Chasman et al. [62]
Consortium name	IHGC	DICE	(–)
*Discovery samples*			
Names	?,?, LUMINA	ERF, NESDA, NTR1, NTR2, Rotterdam study, AGES study	WGHS
Origin	Clinic-based; Finland, Germany, The Netherlands	Population-based; The Netherlands, Iceland	Population-based; US, European descent
# of migraineurs/non-migraineurs	2,731/10,747	2,446/8,534	5,122/18,108
Implicated gene variants (*gene*)^a^	rs1835740 (between *MTDH* and *PGCP*)	rs9908234 (*NGFR*)	rs2651899 (*PRDM16*)
			rs2078371 (–)
			rs10166942 (*TRPM8*)
			rs17172526 (*SEPT7*)
			rs2203834 (*C8orf79*)
			rs13290757 (–)
			rs11172113 (*LRP1*)
*Replication samples*			
Names	?, ?, ?, LUMINA	GEM, NTR3, ATM	GEM, SHIP, IHGC
Origin	Clinic- and population-based/Denmark, Iceland, Germany, The Netherlands	Population-based/The Netherlands, Australia	Population-based/The Netherlands, Germany; clinic-based/Europe
# of migraineurs/non-migraineurs	3,202/40,062	2,957/5,774	3,828/13,949
Replicated gene variants	rs1835740 (between *MTDH* and *PGCP*)	None	rs2651899 (*PRDM16*) rs10166942 (*TRPM8*) rs11172113 (*LRP1*)
Meta-analysis among	Discovery and replication samples	Discovery samples	Discovery and replication samples
Pooled results^a^	rs1835740: OR = 1.18, 95% CI 1.13–1.24, *p* = 1.69 × 10^−11^	rs9908234: *p* = 8.00 × 10^−8^	rs2651899: OR = 1.11, 95% CI 1.07–1.15, *p* = 3.8 × 10^−9^
			rs10166942: OR = 0.85, 95% CI 0.82–0.89, *p* = 5.5 × 10^−12^
			rs11172113: OR = 0.90, 95% CI 0.87–0.93, *p* = 4.3 × 10^−9^
*Other implicated gene variants in discovery samples*			
Significance threshold	*p* ≤ 5 × 10^−5^	*p* < 10^−5^	*p* < 5 × 10^−6^
Gene variants (*genes*)^a^	rs12084862 (*SMYD3*)	31 other SNPs including:	rs2078371 (–)
	rs17528324 (*INSIG2*)	rs11636768 (*AGBL1*)	rs17172526 (*SEPT7*)
	rs17862920 (*TRPM8*)	rs10275320 (*MACC1*)	rs2203834 (*C8orf79*)
	rs2038761 (*MYLK4*)	rs4939879 (*LIPG*)	rs13290757 (–)
	rs6456880 (*ZNF311*)	rs4861775 (*AGA*)	
	rs7753655 (–)	rs986222 (*KIF2OB*)	
	rs10888075 (near *SGCZ*)	rs6107848 (*BMP2*)	
	rs10111769 (–)	rs140174 (*IGLL1*)	
	rs2042600 (*NAV2*)	rs1146161 (*TSPAN2*)	
	rs3794331 (*COG3*)	rs4742323 (*KDM4C*)	
	rs473422 (near *AQP9*)		

*IHGC* International Headache Genetics consortium, *LUMINA* Leiden University Migraine Neuro Analysis, *DICE* Dutch-Icelandic migraine genetics consortium, *ERF* Erasmus Rucphen Family study, *NESDA* The Netherlands Study of Depression and Anxiety, *NTR* The Netherlands Twin Registry, *GEM* Genetic Epidemiology of Migraine, *ATM* Australian Twin Migraine GWA study, *WGHS* Women’s Genome Health Study, *SHIP* Study of Health in Pomerania, *OR* odds ratio, *CI* confidence interval

^a^Results for overall migraine

In the discovery stage, 429,912 genetic variants were genotyped in cases and controls utilizing an Illumina array. Considering a genome-wide significance threshold of *p* ≤ 5 × 10^−8^, only one single nucleotide polymorphism (SNP; rs1835740) was significantly associated with overall migraine. When using a more liberal threshold of *p* ≤ 5 × 10^−5^ eleven SNPs were associated with migraine. The minor allele of rs1835740 was associated with an increased risk for migraine (OR = 1.23, 95% CI 1.15–1.32, *p* = 5.38 × 10^−9^). The population attributable risk for this marker was estimated to be 10.7%. Replication of the association at rs1835740 was successful and the meta-analysis of results from all seven case–control samples indicated an 18% increased risk for overall migraine (OR = 1.18, 95% CI 1.13–1.24, *p* = 1.69 × 10^−11^). In subgroup analyses, the effects were stronger for patients with MA than in those suffering from MO.

Findings from this GWAS lend support for a role of glutamate in the pathophysiology of migraine, in particular MA. rs1835740 is located between the genes *MTDH* and *PGCP*, which are involved in glutamate homeostasis. The study authors have also shown that the minor allele of rs1835740 increases the expression levels of *MTDH* [57]. Further, previous studies in astrocytes have indicated that *MTDH* downregulates *SLC1A2*, which codes for the major glutamate transporter in the brain. This may lead to an increase in the extracellular concentration of glutamate, the major excitatory transmitter in the central nervous system. Glutamate plays a role in trigeminovascular pain processing [58] and may modulate the threshold for cortical spreading depression [59], the putative biological correlate of the aura [60].

In addition to rs1835740, eleven SNPs were associated with migraine with *p* ≤ 5 × 10^−5^ (Table 1). Although missing genome-wide significance, these SNPs may have potential implications for migraine. Nine of these are located within or close to known genes including *SMYD3*, *INSIG2*, *TRPM8*, *MYLK4*, *ZNF311*, *SGCZ*, *NAV2*, *COG3*, and *AQP9*.

The second GWAS was performed by the Dutch-Icelandic (DICE) migraine genetics consortium [61] (Table 1). In the discovery stage, five Dutch and one Icelandic population-based cohorts with 2,446 migraineurs and 8,534 non-migraineurs were investigated. The replication samples derived from two population-based Dutch cohorts and one Australian Twin study totaling 2,957 migraineurs and 5,774 non-migraineurs. Migraine ascertainment in the discovery cohorts was primarily by means of questionnaires, and genotyping was performed utilizing a variety of genotyping platforms. No SNP was associated with migraine at the genome-wide level, 32 SNPs were associated with migraine at *p* < 10^−5^. The best association was seen for rs9908234 (*p* = 8.00 × 10^−8^), which is located in the nerve growth factor (NGF) gene. However, neither this variant nor 18 other chosen variants out of the 32 variants showed associations in the replication cohorts. In a further step, the authors explored associations of previously identified candidate gene variants for migraine with migraine in the pooled discovery dataset. None of the candidates was associated with migraine; however, in gene-based analyses there was modest support for an association of *MTDH*, implicated in the previous GWAS [57], with migraine. Among the reasons potentially explaining the inability to replicate findings from the discovery cohorts are usage of different genotyping platforms, methods of migraine ascertainment, and a lack of power, despite the efforts of bringing together many cohorts.

The third GWAS was also performed to identify common genetic variants for migraine at the population level [62] (Table 1). The study included three population-based cohorts and information from the IHGC. The population-based cohorts were the Women’s Genome Health Study (WGHS), the Genetic Epidemiology of Migraine (GEM) study, and the Study of Health in Pomerania (SHIP). Migraine was either self-reported on questionnaires, diagnosed by headache experts or by a combination of both. Genotyping was performed by Illumina chips (WGHS, IHCG), Affymetrix arrays (SHIP), and iPLEX method from Sequenom (GEM).

The discovery cohort consisted of 23,230 women of European ancestry participating in the WGHS, which is a large population-based cohort in the US. Among those 5,122 women reported migraine, while 18,108 did not. In the genome-wide scan using age-adjusted logistic regression and assuming an additive genetic model, none of the investigated SNPs were associated with migraine at *p* < 5 × 10^−8^. However, seven independent genetic loci had at least one SNP with an association of *p* < 5 × 10^−6^. From each locus the authors selected the most significant SNP (Table 1). None of these seven SNPs was associated with non-migraine headache. In addition, none of the seven SNPs showed associations with migraine in non-additive models, and no other SNP in the genome-wide scan reached genome-wide significance in non-additive models. Finally, association analysis using imputed genotypes of about 2.6 million SNPs identified the same seven loci as found with the genotyped SNPs.

The seven chosen SNPs were evaluated in GEM (774 migraineurs, 942 non-migraineurs), SHIP (306 migraineurs, 2,260 non-migraineurs), and IHGC (2,748 migraineurs, 10,747 non-migraineurs). The effect estimates for three of the SNPs (rs2651899, rs10166942, rs11172113) pointed in the same direction and were comparable in magnitude in all three replication studies as in the WGHS. In the next step a meta-analysis of results for the seven primary SNPs from all four cohorts was performed using a fixed-effects model, since there was no strong evidence of heterogeneity. All three SNPs identified from the replication cohorts based on concordance in direction and magnitude of association were associated with migraine at the genome-wide level (rs2651899: OR = 1.11, 95% CI 1.07–1.15, *p* = 3.8 × 10^−9^; rs10166942: OR = 0.85, 95% CI 0.82–0.89, *p* = 5.5 × 10^−12^; rs11172113: OR = 0.90, 95% CI 0.87–0.93, *p* = 4.3 × 10^−9^). rs2651899 at 1p36.32 is located within the first intron of *PRDM16*, rs10166942 at 2q37 is very close to the transcription start site for *TRPM8*, and rs11172113 at 12q13.3 is in the first intron of *LRP1*. Interestingly *TRPM8* was associated with migraine in the first IHGC GWAS at the sub-genome-wide level [57].

Gender-stratified analyses suggested that the association seen at rs10166942 (*TRPM8*) may be specific for women (meta-regression on gender *p* = 0.004). However, this potential differential association did not appear to be related to genetic variants in the *ESR1* gene, as there was no interaction between rs10166942 (*TRPM8*) and *ESR1* variants. Further investigations looking at potential differences according to migraine aura status and features (for example, unilateral pain location, pulsating pain quality, photophobia, etc.) did not suggest that the associations seen for any of the three variants differed by the presence or the absence of aura or any of the features. Finally, the associations seen for rs2651899 (*PRDM16*) and rs10166942 (*TRPM8*), but not rs11172113 (*LRP1*), were significantly associated with migraine compared to non-migraine headache, underlining the specificity of these variants for migraine.

*TRPM8* codes for a cold and cold-induced burning pain sensor [63] which is primarily expressed in sensory neurons and the dorsal root ganglion [64]. *TRPM8* is investigated as a target in animal models of neuropathic pain [65]. Since migraine and neuropathic pain share some characteristics [66], a role for *TRPM8* in migraine as well as a link between both pain syndromes is plausible.

*LRP1* belongs to the lipoprotein receptor family and is ubiquitously expressed [67]. LRP1 and NMDA glutamate receptors co-localize on neurons and they interact. This is particularly interesting since the previous GWAS by the IHGC has identified a genetic variant involved in glutamate homeostasis [57]. Both findings underline the role of glutamate in migraine pathophysiology and integrate well with pharmacological approaches targeting glutamate receptors for migraine treatment [58].

A role for *PRDM16* in migraine is unknown. Originally identified at a chromosomal breakpoint associated with myelodysplastic syndrome and leukemia [68], more recent research has focused on its role in brown fat development [69].

## Implications

Endeavors to decipher the genetics of migraine have been successful with respect to FHM; however, there is ongoing debate about whether the genes involved (all coding for ion channels) are specific for migraine headache, the accompanying hemiplegic aura, or possibly for both. In contrast, attempts for common forms of migraine, although multifold, were often ambiguous. Positive results from candidate gene association studies have mostly not been robust, with the exception, for example, for the *MTHFR* 677C<T polymorphism, which shows an association with migraine in pooled analysis of the available studies. Many other genetic variants were not associated with migraine or replication of positive results is either missing or failed. Linkage studies produced some encouraging results, but it remains unclear which genes under the linkage peaks may be important for migraine. Reasons hampering an unequivocal success of previous studies include limited sample size, heterogeneity of the migraine phenotype as well as study design and conduct.

Migraine GWASs have overcome some of these drawbacks and advanced our understanding of migraine genetics with the following implications. First, available GWASs have identified four new genetic variants that are associated with migraine with genome-wide significance and none of the implicated genes has been a candidate in previous genetic studies. Second, the role for three of the variants may plausibly be integrated with current concepts of migraine pathophysiology. rs1835740 modulates glutamate homeostasis [57] and rs11172113 (*LRP1*) [62] may also impact glutamate pathways through interaction of LRP1 receptors with NMDA glutamate receptors. Further, the association at rs10166942 (*TRPM8*) for the first time implicates a gene in a pain related pathway in migraine. Third, the available results are compatible with shared pathophysiology among common forms of migraine, since associations for rs2651899 (*PRDM16*), rs10166942 (*TRPM8*), and rs11172113 (*LRP1*) did not differ for MA and MO [62]. Fourth, the results also suggest differences between population-based and clinic-based forms of migraine. While rs2651899 (*PRDM16*), rs10166942 (*TRPM8*), and rs11172113 (*LRP1*) appear most important for common migraine (i.e., at the population level) [62], rs1835740 may play the major role in more severe forms of migraine (i.e., clinic-based populations) [57]. This agrees with the fact that virtually all migraineurs in the IHGC study suffered from MA [57], which is often considered more severe than MO. However, the association of rs1835740 with MA may also underline the importance of glutamate for the aura phenotype among migraineurs. Finally, the effect estimates indicate that each of the variants only confers a small to moderate change in risk for migraine (Table 1). This agrees with migraine being a heterogeneous disorder, where many gene variants work together to produce the migraine phenotype.

## Outlook

Despite enormous efforts, the published GWASs can “only” claim to have identified four genetic variants to be associated with migraine. This number appears small. However, it is imperative to keep in mind the broad clinical phenotype of migraine, which complicates the identification of genetic variants. In addition, we must consider that the implicated genes are both new, replicated in independent samples, and at the same time plausibly involved in migraine pathophysiology.

Additional approaches in studying migraine genetics include focusing on endophenotypes of migraine and investigating larger numbers of migraineurs and non-migraineurs. Trait component analysis and latent class analysis have been used previously [44, 55, 56], and may also be promising approaches for future GWASs. Further, large international collaborations are ongoing aiming at combining genome-wide genetic data on migraine to increase the power to identify additional genetic variants in migraine. Additional variants will likely be identified. Finally, we need to understand that GWASs “only” investigate hundreds of thousands of common and known gene variants. However, there is ample space in the genome that is not investigated. Hence, variants identified may only be markers for migraine, which are in linkage disequilibrium with the causative variants. Further, there may be areas in the genome that are not well represented on the GWAS platforms, but which may plausibly harbor variants involved in migraine pathophysiology. In both scenarios it may be useful to utilize DNA sequencing methods to investigate certain genes or chromosomal regions to obtain a complete map of all variants, including rare variants, in these regions. This may facilitate the discovery of the single best marker in a region or even identify new variants. Considering the large number of DNA base pairs to be sequenced as well as the large number of individuals that may have to investigated, high-throughput sequencing methods may have to be used. However, the limitations associated with each of the available platforms need to be considered and the technology used must be tailored to the specific projects.
